# Immunohistochemical assessment of PD-L1 expression using three different monoclonal antibodies in triple negative breast cancer patients

**DOI:** 10.1007/s00404-022-06529-w

**Published:** 2022-04-04

**Authors:** Gilda Schmidt, Margit Maria Guhl, Erich-Franz Solomayer, Gudrun Wagenpfeil, Mohammed Eid Hammadeh, Ingolf Juhasz-Boess, Jan Endrikat, Mariz Kasoha, Rainer Maria Bohle

**Affiliations:** 1 Department of Gynecology, Obstetrics and Reproductive Medicine, University Medical School of Saarland, Kirrberger Straße, 66421 Homburg/Saar, Germany; 2 Institute of General and Special Pathology, University Medical School of Saarland, 66421 Homburg, Germany; 3 Institute of Medical Biometry, Epidemiology and Medical Informatics, Saarland University, 66421 Homburg, Germany; 4 Department of Gynecology, Obstetrics and Reproductive Medicine, University of Freiburg Faculty of Medicine, 79085 Freiburg im Breisgau, Germany

**Keywords:** Triple-negative breast cancer, PD-L1 expression, Monoclonal antibodies 22C3, 28-8 and SP142

## Abstract

**Background:**

PD-L1 receptor expression in breast cancer tissue can be assessed with different anti-human PD-L1 monoclonal antibodies. The performance of three specific monoclonal antibodies in a head-to-head comparison is unknown. In addition, a potential correlation of PD-L1 expression and clinico-pathological parameters has not been investigated.

**Methods:**

This was a retrospective study on tissue samples of patients with histologically confirmed triple negative breast cancer (TNBC). PD-L1 receptors were immune histochemically stained with three anti-human PD-L1 monoclonal antibodies: 22C3 and 28-8 for staining of tumor cell membranes (TC) and cytoplasm (Cyt), SP142 for immune cell staining (IC). Three different tissue samples of each patient were evaluated separately by two observers in a blinded fashion. The percentage of PD-L1 positive tumor cells in relation to the total number of tumor cells was determined. For antibodies 22C3 and 28-8 PD-L1 staining of 0 to < 1% of tumor cells was rated "negative", 1–50% was rated "positive" and > 50% was rated "strong positive". Cyt staining was defined as “negative” when no signal was observed and as “positive”, when any positive signal was observed. For IC staining with SP142 all samples with PD-L1 expression ≥ 1% were rated as “positive”. Finally, the relationship between PD-L1 expression and clinico-pathological parameters was analyzed.

**Results:**

Tissue samples from 59 of 60 enrolled patients could be analyzed. Mean age was 55 years. Both the monoclonal antibodies 22C3 and 28-8 had similar properties, and were positive for both TC in 13 patients (22%) and for Cyt staining in 24 patients (40.7%). IC staining with antibody SP142 was positive in 24 patients (40.7%), who were also positive for Cyt staining. The differences between TC and Cyt staining and TC and IC staining were significant (*p* = 0.001). Cases with positive TC staining showed higher Ki67 expression compared to those with negative staining, 40 vs 30%, respectively (*p* = 0.05). None of the other clinico-pathological parameters showed any correlation with PDL1 expression.

**Conclusions:**

Antibodies 22C3 and 28-8 can be used interchangeably for PD-L1 determination in tumor cells of TNBC patients. Results for Cyt staining with 22C3 or 28-8 and IC staining with SP142 were identical. In our study PD-L1 expression correlates with Ki67 expression but not with OS or DFS.

## Introduction

Triple negative breast cancer (TNBC) is a subtype of breast cancer (BC) defined by total lack of hormone receptor expression, i.e., estrogen receptor negative (ER−), progesterone receptor negative (PR−) and human epidermal growth factor receptor negative (Her2neu−). TNBC affects about 15–20% of women with BC [15, 23]. Compared to other subtypes, TNBC is the most malignant form of BC [7] with early recurrence and early distant metastases [17] resulting in a poor prognosis [15, 16].

The median overall survival (OS) of metastatic TNBC patients is about 10.2 months [1]. So far, chemotherapy is the treatment of choice as hormone or antibody therapy lack a suitable target [14].

Tumor cells expressing PD-L1 on their cell surface can inactivate immigrating cytotoxic T cells and thus evade destruction [18]. New studies on “immune checkpoint inhibitors” block either PD-L1 receptors on the tumor cell or PD-1 receptors on the T cell and consequently allow immune cells to attack [10].

On March 2019, the Food and Drug Administration (FDA) approved the immune checkpoint inhibitor atezolizumab in combination with protein-bound paclitaxel (nab-paclitaxel) for TNBC patients who express PD-L1 > 1%. In the registration study, “Impassion 130” PD-L1 expression was determined with anti-PD-L1 monoclonal antibody SP142 [22]. By inhibiting PD-L1, atezolizumab enables the activation of T cells and thus restores their ability to recognize and destroy tumor cells [11].

The aim of this study was to compare the ability of three different anti-human PD-L1 monoclonal antibodies to show PD-L1 expression in tumor and immune cells in women with TNBC. In addition, a correlation between PD-L1 expression and clinico-pathological parameters was explored.

## Materials and methods

### Study design

This retrospective study included women with histologically confirmed TNBC treated at the Department of Gynecology and Obstetrics, Saarland University Medical Center, Homburg/Saar, Germany between the end of 2004 and mid-2013. All patients received surgery but not neoadjuvant chemotherapy. All study data were retrieved from the medical records. The Ethics Committee of Saarland, Germany approved this study.

### Tissue sample preparation

All formalin-fixed paraffin-embedded (FFPE) tissue blocks were retrieved from the archive of our institute of pathology. The FFPE blocks were cut into 4 µg thick tissue slices and stained by hematoxylin–eosin (H&E) staining. An experienced pathologist with more than 30 years of experience analyzed these sections and defined representative tumor areas for tissue microarray (TMA) construction.

### TMA preparation

It is prepared from each TMA block for H&E- and immunohistochemical (IHC) staining. The pathologist determined the most meaningful areas within each tissue block, sampled three 1 mm cores from each block using a biopsy needle and placed them into three separate micro tubes. These tubes were sent to Zytomed Systems GmbH in Berlin, Germany, for preparation of the TMA blocks, producing a total of 60 cores (from 20 cases with 3 cores each). Finally, four consecutive serial 4 μm sections were prepared from each TMA block to be used for H&E- and immunohistochemical (IHC) staining.

### IHC staining

Three anti-human PD-L1 antibodies were used: antibodies 22C3 and 28-8 for staining of membranes and cytoplasm of tumor cells and SP142 for immune cell staining (Table 1).

**Table 1:** Antibodies used in this study

Primary antibody	Antibody	Vendor	Dilution	References
Anti-human PD-L1	22C3	Dako	1:50	Dako (2016) Monoclonal Mouse anti-human PD-L1 antibody 22C3. Dako Produktdatenblatt. P04435EFG_01(M3653). p 1–4
Anti-human PD-L1	28-8	Abcam	1:200	abcam, Anti-PD-L1 antibody (28-8) abcam Product datasheet, 2015. Q9NZQ7(ab205921). p 1–11
Anti-human PD-L1	SP142	Dako	–	Ventana Medical Systems, I., VENTANA PD-L1 (SP142) Assay. Ventana Produktdatenblatt, 2018. 1018007DE RevA(FT0700-410p). p 1–11

All IHC staining steps were performed at our institute of pathology using an automated staining tool (BenchMark XT, Ventana Medical System, Inc., Tucson, AZ, USA) and two different detection kits: UltraView Universal Alkaline Phosphatase Red kit (antibody 22C3 and 28-8) and OptiView DAB IHC Kit for (antibody142) (Ventana Medical Systems).

After staining, slides were washed under running tap water at approx. 50 °C, hydrated in a series of graded alcohols, rinsed in xylene and finally covered with a mounting medium and a cover glass.

### Documentation and evaluation of IHC stained slides

#### Documentation

Slides stained with anti-PD-L1 antibody 22C3 and antibody 28-8 were scanned using Nikon Supercoolscan ED 5000 scanner to record an overview of the staining. Then, individual cores were analyzed under a Zeiss microscope (Axioskop 40, Carl Zeiss, Germany), and selected images were recorded with a digital camera (AxioCam MRC, Carl Zeiss, Germany) using Axiovision Documentation Rel.4.8 program. Photo documentation of the slides stained with anti-PD-L1 antibody 142 was done using the sacn service from Sysmex (Norderstedt, Germany). Images of scanned sections were later downloaded, viewed, evaluated and documented in various magnifications via the Sysmex Case Viewer (viewer software 3DHistech Case Viewer).

#### Observers

Three different samples of each patient were evaluated separately by two observers in a blinded fashion. One was a pathologist from our institution with more than 30 years of experience and the second observer had completed a special training course at the Qualitätssicherungs-Initiative Pathologie GmbH (QuiP, Berlin, Germany).

#### Quality and evaluation criteria

Valid cases had to show at least 100 evaluable/vital tumor cells, damaged or necrotic cells were excluded.

Staining of anti-PD-L1 antibodies 22C3 and 28-8 was evaluated in the tumor cell cytoplasma (Cyt) and cell membrane (TC)*.* Membranous staining was recorded as positive if at least one red (i.e., positive) signal could be recorded, irrespective of extent or intensity [20].

**Fig. 1:**
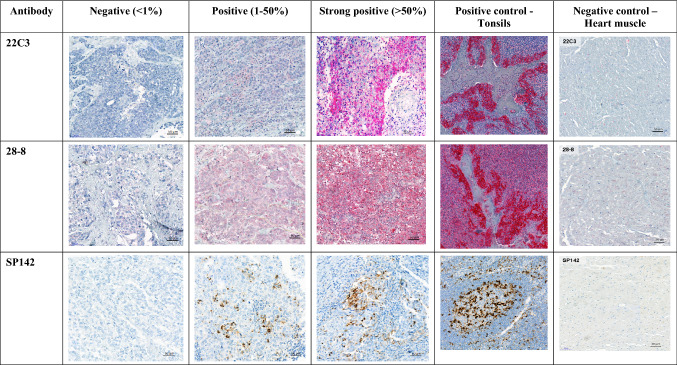
Immunhistochemical staining of tumor tissue and controls with three anti-PD-L1 monoclonal antibodies

The percentage of PD-L1 positive tumor cells in relation to the total number of tumor cells was scored as follows: PD-L1 expression 0 to < 1% was rated "negative", 1–50% was rated "positive" and a PD-L1 expression > 50% was rated "strong positive". Cyt staining was defined as “negative” when no signal was observed and as “positive” when any positive signal was observed (Fig. 1).

In addition, IC staining with antibody SP142 was assessed. Here, individually distributed immune cells, punctiform immune cell and immune cell aggregates in the intra-tumoral stroma were evaluated [24]. Thus, all tumor areas covered by PD-L1 in immune cells were evaluated. Again, the percentage of PD-L1 positive immune cells was calculated as a percentage of the counted tumor cells. Membranous staining of tumor cells was not taken into account. All samples with PD-L1 expression ≥ 1% were evaluated as PD-L1 “positive” [21].

In every staining run, tonsil tissue and heart muscle served as positive and negative controls, respectively (Fig. 1).

The results of the individual samples were combined into one result. If the individual percentages from one patient were heterogeneous, and the percentage distribution (based on 100 vital tumor cells) in the total tumor mass was evaluated.

Finally, the relationship between PD-L1 expression and clinical parameters was analyzed.

### Statistical analysis

Descriptive analysis was applied using absolute and relative frequencies for categorical variables and mean, median and range for continuous variables. McNemar-test was used for testing the association between dependent categorical variables. For group comparisons of quantitative data we used the Mann–Whitney *U* test assuming non-normality. Data were analyzed using SPSS version 25. A two-sided *p* value of 5% or lower was considered statistically significant. We did not correct for the issue of multiple testing due to the explorative nature of the study.

## Results

### Study population

Sixty patients were included in this study, one was excluded because of a lack of tumor cells in the tissue sample. Mean follow-up was 92 months (range 0–153 months).

Mean age was 55 years, 54 patients (90%) had an invasive ductal carcinoma, 40 patients (66%) had G3 cancers and 59 (98.3%) had no metastases (M0) (Table 2).

**Table 2:** Clinico-pathological parameters of study population (*N* = 60)

Clinicopathological characteristic	
Age at disease diagnosis (mean/range) years	55 (29–91)
History of previous tumor	*N* (%)
No	56 (93.3)
Yes^a^	4 (6.7)
Histology	
Invasive ductal	54 (90)
Other^b^	6 (10)
Grading	
G1/G2	20 (33.3)
G3	40 (66.7)
T status	
T1	31 (51.7)
T2	24 (40)
T3	3 (5)
T4	2 (3.3)
N status	
N0	44 (73.3)
N1/N2/N3	16 (26.7)
M status	
M0	59 (98.3)
M1^c^	1 (1.7)
Ki67%	
≤ 14%	6 (10)
> 14%	44 (73.3)
Unknown	10 (16.7)
HER2	
0	31 (51.7)
1	27 (45)
2	2 (3.3)
Disease free survival (DFS)	
Mean follow-up 92 months (range: 0–153 months)	
No	42 (70)
Yes	18 (30)
Death	
Mean follow-up 92 months (range: 0–153 months)	
No	42 (70)
Yes	18 (30)

^a^Ovarian cancer (*N* = 1), endometrial cancer (*N* = 1), breast cancer (HER positive) (*n* = 1), and thyroid cancer (*n* = 1)

^b^Invasive lobular (*N* = 3), tubulo-lobular (*N* = 1), multicentral (*N* = 1), and invasive papillary (*N* = 1)

^c^Bone metastases

### PD-L1 expression

Anti-PD-L1 antibody 22C3 and 28-8 showed identical results for TC staining (46, 12, and 1 case for negative, positive, and strong positive staining, respectively) as well as for Cyt staining (35 and 24 cases for negative and positive staining, respectively).

IC staining with anti-PD-L1 antibody SP142 was negative in 35/59 cases and positive in 24/59 patients (Table 3).

**Table 3:** Protein expression patterns of PD-L1 in tumor and immune cells with 3 monoclonal antibodies (*N* = 59)

PD-L1 antibody	Membranous staining (TC)			Cytoplasmic staining (Cyt)		Immuno cells staining (IC)	
	Negative (< 1%)	Positive (1–50%)	Strong positive (> 50%)	Negative	Positive	Negative (< 1%)	Positive (> 1%)
22C3	46 (78%)	12 (20.3)	1 (1.7%)	35 (59.3%)	24 (40.7%)	–	–
28-8	46 (78%)	12 (20.3)	1 (1.7%)	35 (59.3%)	24 (40.7%)	–	–
SP142	–	–	–	–	–	35 (59.3%)	24 (40.7%)

*TC* Tumor cell, *Cyt* Cytoplasma, *IC* Immune cell

### PDL1 staining patterns

Positive staining with antibodies 22C3 and 28-8 was significantly higher in Cyt compared to TC, 24/59 patients (40.7%) vs 13/59 (22%) (*p* = 0.001). ICs stained with antibody SP142 showed the same positive staining patterns as Cyt, i.e., 24/59 patients (40.7%) (*p* ≥ 0.05). Thus, the difference between TC staining and IC staining was also significant (*p* = 0.001).

**Fig. 2:**
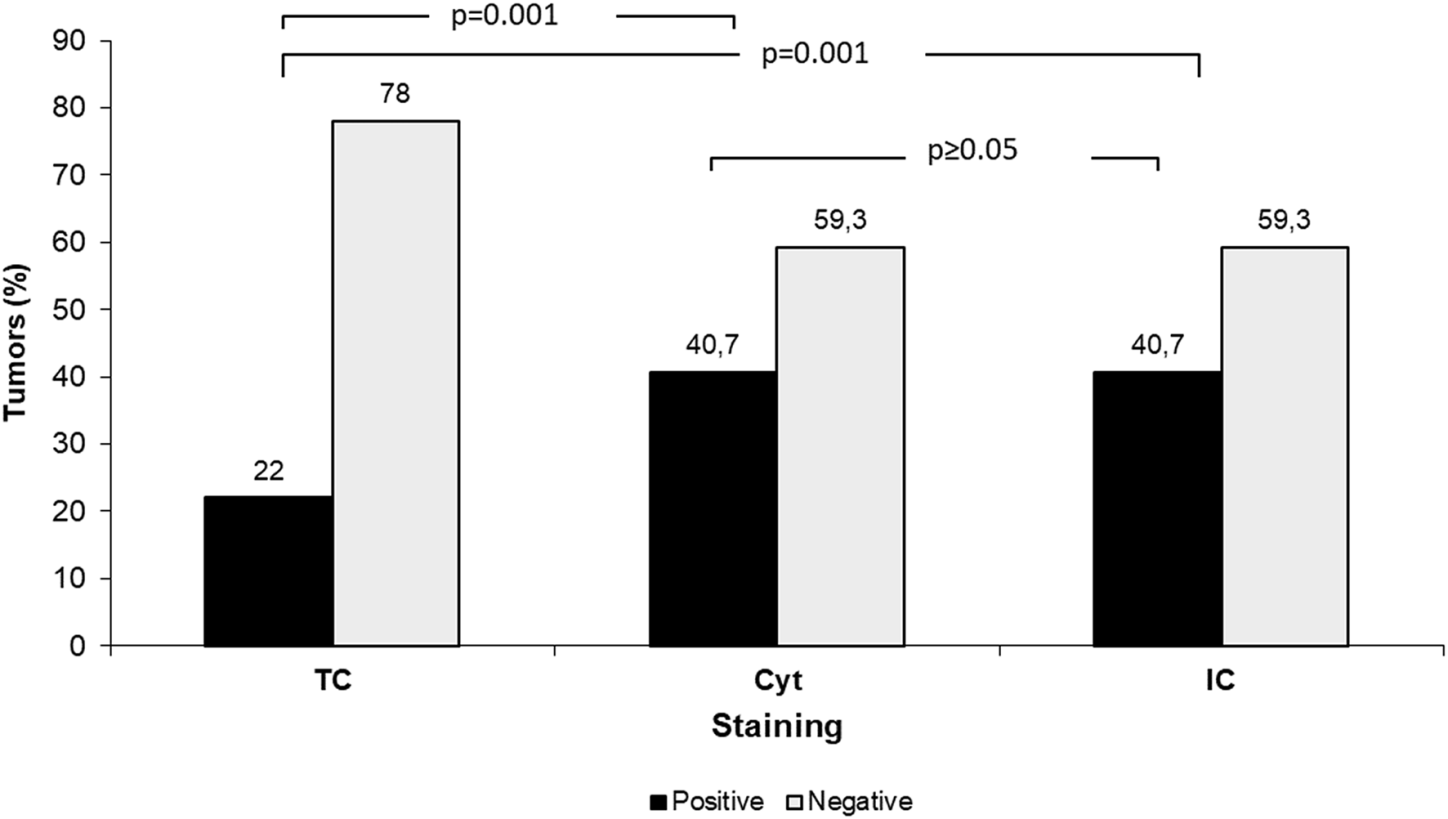
Staining patterns of tumor cell membranes (TC), tumor cell cytoplasm (Cyt) and immune cells (IC) with either antibody 22C3 and 28-8 combined or SP142. *N* = 59. TC and Cyt staining assessed with antibodies 22C3 and 28-8. IC staining assessed with antibody SP142

All cases with positive TC staining showed also positive staining in Cyt and IC. The cases with positive staining in Cyt and IC were identical. A correlation between Cyt staining with antibodies 22C3 and 28-8 vs IC staining with antibody SP142 has been proven (Fig. 2).

### PD-L1 expression vs clinico-pathological characteristics

**Fig. 3:**
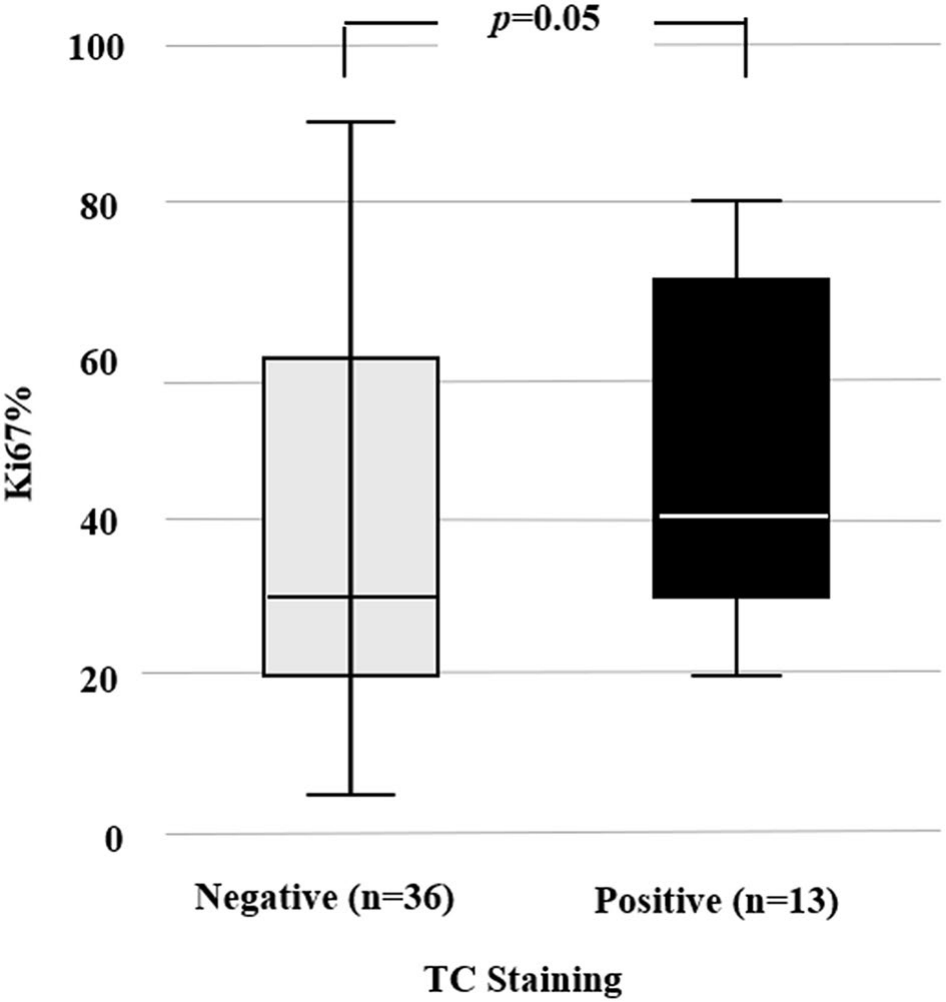
Mean Ki67 expression by TC staining patterns of PD-L1 using antibodies 22C3 and 28-8

Cases with positive TC staining showed significantly higher Ki67 expression compared to those with negative staining, 40 vs 30%, respectively (*p* = 0.05) (Fig. 3).

None of the other clinico-pathological parameters shown in Table 2, including OS/PFS, showed any correlation with PDL1 expression.

## Discussion

This study compared the ability of three different anti-human PD-L1 monoclonal antibodies to show PD-L1 expression in tumor and immune cells of women with TNBC. Monoclonal antibodies 22C3 and 28-8 can be used interchangeably for assessing PD-L1 expression on tumor cells. Results for Cyt staining with 22C or 28-8 and IC staining with SP142 were identical. No correlation between PD-L1 expression and clinico-pathological parameters was found.

PD-L1 expression assessed with antibodies 22C3 and 28-8 showed similar results for TC and Cyt staining. However, this result is only valid for breast cancer tissue, in particular valid for patients with TNBC. This may not apply to other tumor entities. However, results on non-small-cell lung cancer were similar [12, 20].

The German harmonization study Scheel et al. [20] also showed for non-small cell lung cancer (NSCLC) that the anti-PD-L1 antibodies clone 22C3 and clone 28-8 provide comparable results. In the international blueprint study [12], the staining results for NSCLC were also consistent with antibody clones 22C3 and 28-8. While clone SP142 showed divergent results in both studies.

In urothelial carcinoma clone 22C3 and SP142 showed strong agreement in TC and IC. Both clones are potentially useful in the evaluation of PD-L1 expression in urothelial carcinoma [19].

While this study could not prove any correlation of PD-L1 expression with survival, Huang et al. showed in a meta-analysis on 14,367 BC patients that PD-L1 expression on tumor cells associates with high-risk clinico-pathological parameters and poor prognosis. However, PD-L1 in combination with tumor infiltrating lymphocytes may relate to significantly longer DFS (*p* = 0.001) and OS (*p* < 0.0001). Comprehensive assessment of TCs and TILs is required when evaluating the clinical relevance of PD-L1 expression in future studies [13].

While no correlation between IC staining and clinico-pathological parameters could be demonstrated, Schmid et al. showed in a large phase III study, that metastatic TNBC patients with PD-L1 expression of ≥ 1% in ICs benefit from therapy with the checkpoint inhibitor atezolizumab in combination with nab-paclitaxel. Schmid et al. reported an improvement in progression-free survival of 5 versus 7.5 months (HR 0.62, *p* < 0.0001) and in OS of 15.5 vs. 25 months [22].

Similarly, Broekhoff et al. reported longer DFS and OS in 103 TNBC patients with PD-L1 expression. They state that PD-L1 expression indicates an enhanced immunological anti-tumor activity resulting in favorable prognosis [2].

Finally, Reis et al. showed in urothelial carcinoma that PD-L1 expression in ICs has a higher predictive value than PD-L1 expression in TCs (*R* values: 0.901–0.780) [19].

### Correlation of PD-L1 expression and clinico-pathological parameters

Although PD-L1 expression is considered a clinically relevant prognostic parameter [9, 21], no correlation between PD-L1 expression and clinico-pathological parameters was found in this study. In contrast, Gluz et al. recently published a sub-analysis of the ADAPT study showing TNBC patients with high PD-L1 expression had a higher pCR rate (pathological complete remission, *p* < 0.05) and also a significant longer OS [9]. Notably, one patient in this study with PD-L1 expression of > 50% was reported to be disease-free for over 10 years.

However, there was one exception: patients with positive TC staining showed significantly higher Ki67 expression compared to those with negative staining, 40 vs 30%, respectively (*p* = 0.05) (Fig. 3). Both Ki67 and TC staining indicate a higher malignancy of the tumor. These results confirm the studies by Doğukan et al. [8] and Huang et al. [13].

### Limitations

The following limitations need to be addressed: (1) the cohort of 59 valid cases was small, Ki67 could be determined in 49 patients only; (2) it is unknown whether the cores taken for the tissue sample were representative for the whole tumor, in particular in highly heterogenous cancers.

### Conclusions

Antibodies 22C3 and 28-8 can be used interchangeably for PD-L1 determination in TBNC patients. Results for Cyt staining with 22C or 28-2 and IC staining with SP142 were identical. In our study, PD-L1 expression correlates with Ki67 expression but not with OS or DFS.
